# The autonomic nervous system: A potential link to the efficacy of acupuncture

**DOI:** 10.3389/fnins.2022.1038945

**Published:** 2022-12-08

**Authors:** Yan-Wei Li, Wei Li, Song-Tao Wang, Yi-Nan Gong, Bao-Min Dou, Zhong-Xi Lyu, Luis Ulloa, Shen-Jun Wang, Zhi-Fang Xu, Yi Guo

**Affiliations:** ^1^Research Center of Experimental Acupuncture Science, Tianjin University of Traditional Chinese Medicine, Tianjin, China; ^2^School of Acupuncture & Moxibustion and Tuina, Tianjin University of Traditional Chinese Medicine, Tianjin, China; ^3^National Clinical Research Center for Chinese Medicine Acupuncture and Moxibustion, Tianjin, China; ^4^Department of Anesthesiology, Center for Perioperative Organ Protection, Duke University, Durham, NC, United States

**Keywords:** acupuncture, autonomic nervous system, sympathetic nerve, parasympathetic nerve, neuronal circuit

## Abstract

The autonomic nervous system (ANS) is a diffuse network that regulates physiological systems to maintain body homeostasis by integrating inputs from the internal and external environment, including the sympathetic, parasympathetic, and enteric nervous systems (ENS). Recent evidence suggests that ANS is one of the key neural pathways for acupuncture signal transduction, which has attracted worldwide attention in the acupuncture field. Here, we reviewed the basic and clinical research published in PubMed over the past 20 years on the effects of acupuncture on ANS regulation and homeostasis maintenance. It was found that acupuncture effectively alleviates ANS dysfunction-associated symptoms in its indications, such as migraine, depression, insomnia, functional dyspepsia, functional constipation. Acupuncture stimulation on some specific acupoints activates sensory nerve fibers, the spinal cord, and the brain. Using information integration and efferents from a complex network of autonomic nuclei of the brain, such as the insular cortex (IC), prefrontal cortex, anterior cingulate cortex (ACC), amygdala (AMG), hypothalamus, periaqueductal gray (PAG), nucleus tractus solitarius (NTS), ventrolateral medulla (VLM), nucleus ambiguus (AMB), acupuncture alleviates visceral dysfunction, inflammation *via* efferent autonomic nerves, and relieves pain and pain affect. The modulating pattern of sympathetic and parasympathetic nerves is associated with acupuncture stimulation on specific acupoints, intervention parameters, and disease models, and the relationships among them require further exploration. In conclusion, ANS is one of the therapeutic targets for acupuncture and mediates acupuncture’s actions, which restores homeostasis. A systemic study is needed to determine the rules and mechanisms underlying the effects of acupoint stimulation on corresponding organs mediated by specific central nervous networks and the efferent ANS.

## Introduction

Acupuncture forms an integral part of traditional Chinese medicine, and its curative effects have been demonstrated in clinical practice for thousands of years. According to the World Health Organization (WHO) Traditional Medicine Strategy: 2014–2023, acupuncture is widely used in 183 countries and regions, and many high-quality clinical studies have verified its efficacy in different applications (Lu et al., 2022). However, knowledge of the scientific process underlying acupuncture’s activities is critical for determining and expanding its applicability. Overall, the proposed mechanisms can be divided into three processes, namely, “sensation,” “transmission,” and “effect.” “Sensation” represents the local perception of acupuncture stimulation at acupoints and the conversion of the sensation to transmissible information. The microenvironments of acupoints share characteristics but differ depending on the acupoint and the intervention parameters of acupuncture stimulation. “Effect” represents the response in the target organs and cells, which varies according to different diseases. Neural pathways are crucial to “transmission,” which is the transfer of acupuncture signals from acupoints to their intended target organs (Longhurst, 2010). Acupuncture activates peripheral afferent nerves, transmitting sensory information from the spinal cord to the brain, where it is integrated. The target organs may be modified due to activating efferent nerves or neuro-endocrine pathways (Torres-Rosas et al., 2014; Liu S. et al., 2020; Liu et al., 2021; Ulloa, 2021, 2022). However, the mechanisms underlying information integration in the brain and the outgoing pathways are not fully understood.

The autonomic nervous system (ANS) is a diffuse network that regulates physiological systems by integrating inputs from the internal and external environment to maintain body homeostasis. The ANS consists of the central autonomic nervous network (CAN) and two major efferent components, the sympathetic and parasympathetic nervous systems (SNS and PNS, respectively), and the peripheral semiautonomous neural network known as the third branch of the ANS, namely, the enteric nervous system (ENS). The distribution of the ANS in the peripheral and central nervous systems is the basis for the precise regulation of relatively balanced and rhythmic visceral activities, such as body temperature, respiration, heartbeat, digestion, excretion, and immune functions, to regulate the body’s metabolism (Wehrwein et al., 2016). For instance, the ANS regulates the immune system by innervating immune organs such as the spleen, thymus, and lymph nodes and regulates the gastrointestinal tract, bladder, heart, and other organs by releasing neurotransmitters such as acetylcholine and norepinephrine that contribute to the maintenance of homeostasis (Nance and Sanders, 2007).

Various lines of evidence have shown that acupuncture alleviates diseases and symptoms accompanied by autonomic disorders, such as migraine, depression, insomnia, functional dyspepsia, and constipation. An increasing number of studies have also demonstrated that acupuncture can regulate blood pressure, pupil size, skin conductance, skin temperature, heart rate (HR) and/or pulse rate, and heart rate variability (HRV) mediated by the ANS. For instance, visceral sympathoexcitatory reflexes induced by stimulation of the gallbladder with bradykinin are attenuated by electroacupuncture at *Jianshi* (PC5) and *Neiguan* (PC6) located over the median nerve (Zhou et al., 2007). Liu S. et al. (2020) recently reported how electroacupuncture activates district autonomic networks to control inflammation in somatotopy-, stimulation intensity-, and disease state-dependent manner. However, the underlying mechanism is complex and concerns diverse organs and systems.

Here, we review the basic and clinical studies published over the past 20 years in PubMed on the role of the ANS in acupuncture and we further discuss how acupuncture parameters affects the ANS, including the complex network of autonomic nuclei and efferent peripheral networks controlling organ functions. These studies provide anatomical basis for the clinical application of acupuncture.

## Acupuncture is effective in alleviating autonomic nervous system dysfunction-associated symptoms in its indications

According to a 2018 US survey, acupuncture is widely used to treat its indications of which the top 10 are lower back pain (LBP), depression, anxiety, headache, arthritis, allergy, systemic pain, female infertility, insomnia, and shoulder and neck pain, with pain ranking first among them. Further indications include mental health management of mood disorders, immune system dysfunction, and gastrointestinal, gynecological, and neurological diseases (Wang et al., 2018). The majority of these disorders are associated with or accompanied by ANS dysfunction.

Lower back pain, shoulder and neck pain, and systemic and visceral pain are associated with ANS dysfunction. Koenig and colleagues evaluated ANS activity in patients with chronic low back pain (CLBP), observing higher HR and dermal electrical activity, together with low-frequency HRV compared with controls, indicating increased systemic SNS activity in patients with CLBP (El-Badawy and El Mikkawy, 2016; Koenig et al., 2016). Patients with cervical and shoulder pain and fibromyalgia have been shown to have excessive sympathetic activity and reduced parasympathetic activity. Increased sympathetic activity has been found to exacerbate myofascial trigger points for spontaneous local pain in patients with chronic neck and shoulder pain (Hallman et al., 2011). Visceral hypersensitivity is an essential factor underlying abdominal pain in functional gastrointestinal disorders such as irritable bowel syndrome (IBS), and visceral hypersensitivity related to dysfunction of the ANS. Increased SNS activity and decreased PNS activity are the most frequently noted differences when IBS patients are compared with healthy controls (Manabe et al., 2009; De Winter et al., 2016).

Acupuncture analgesia is well-known for its efficacy, and pain relief by acupuncture is accompanied by ANS activity regulation. For example, several randomized controlled trials (RCTs) have demonstrated the long-term efficacy of acupuncture in preventing migraine, and the low-frequency HRVs in migraine patients were significantly reduced during electroacupuncture at *Fengchi* (GB20), *Shuaigu* (GB8) and other acupoints treatment, suggesting that acupuncture mitigates migraine accompanied with the regulation of the ANS (Zhao et al., 2017). Using a non-invasive cardiovascular autonomic function test, it was found that electroacupuncture at *ashi* points in the lower back resulted in analgesia, restored reduced vagal tone, and increased sympathetic activity in patients with CLBP (Shankar et al., 2011).

Numerous psychological disorders, such as depression and anxiety, have been linked to the ANS, and HRV is widely used in these conditions as a marker of abnormal ANS activity. For instance, a meta-analysis of 13 rigorous cross-sectional studies, including 312 depressed and 374 non-depressed patients, showed an association between a diagnosis of depression and mild-to-moderate changes in cardiac vagal control, with resting vagal control being significantly reduced in depressed patients compared with non-depressed patients (Rottenberg, 2007). A meta-analysis of 18 studies, including 673 depressed and 407 healthy people, found a negative correlation between depression severity and HRV (Kemp et al., 2010).

It has been found that press needle stimulation at *Ximen* (PC4), *Shousanli* (LI10), *Yinlingquan* (SP9), and *Sanyingjiao* (SP6) could improve Beck’s Depression Inventory scores, systolic/diastolic blood pressure, and the coefficient of variation between the R-R intervals of depressed patients, suggesting that acupuncture treatment for depression accompanied with the increase of vagal activity (Noda et al., 2015). It was found that transcutaneous auricular vagus nerve stimulation (taVNS) can reduce the symptoms of depression, such as anxiety, cognitive impairment, sleep disturbance, and feelings of hopelessness. The default mode network (DMN), central executive network (CEN), and the salience network (SN) are the three most important intrinsic networks in the human brain. Alterations within and between the DMN, CEN, and SN have been identified as “hotspots” in major depressive disorder (MDD). taVNS can decrease the connectivity between the posterior DMN and the emotional and reward circuits and increase the connection between the anterior and posterior DMN, between the anterior DMN and the CEN, and between the CEN and the emotional and reward circuits in MDD patients (Liu C. H. et al., 2020). Auricular acupuncture works primarily by activating the vagus nerve. However, more research is needed to determine whether auricular acupuncture helps treat MDD.

Evidence has shown that gastrointestinal diseases are accompanied by ANS dysfunction (Zhang S. et al., 2018). By detecting 24-h HRV, gastrointestinal motility, and gastric acid secretion, Tominaga et al. (2016) found that patients with functional dyspepsia had increased sympathetic tone and decreased vagal reactivity and that patients with ANS disorders had more serious gastrointestinal symptoms with higher indigestion scores. According to multiple meta-analyses, acupuncture is a valuable option for individuals who are unsuitable for prokinetic therapy and experience fewer side effects than gastrointestinal motility drugs used to treat functional dyspepsia (Ho et al., 2017). A typical gastrointestinal disorder is functional constipation. Percutaneous electrical stimulation at ST36 reduces the cumulative incidence of constipation after apoplexy, increases the number of weekly and spontaneous defecations, reduces defecation strain, improves stool consistency, increases vagal nerve activity, and reduces sympathetic nerve activity (Liu et al., 2018). Another RCT on the treatment of functional constipation found that electroacupuncture ST25, *Fujie* (SP14), and *Shangjuxu* (ST37) could increase the number of entirely spontaneous defecations in patients with functional constipation while decreasing the prevalence of adverse effects (Liu Z. et al., 2016), but whether these effects are related to ANS regulation requires further exploration.

To summarize, it can be seen from the above studies that acupuncture can alleviate many clinical conditions as acupuncture can address ANS dysfunction, and the regulation of ANS activity accompanies the therapeutic effects of acupuncture on these diseases.

## Autonomic nervous system

The CAN consists of a set of interconnected regions in the telencephalon, diencephalon, and brainstem, including the insular cortex (IC), anterior cingulate cortex (ACC), amygdala (AMG), hypothalamus, periaqueductal gray (PAG), parabrachial nuclei, dorsal vagal complex (DVC), ventrolateral medulla (VLM), and the nucleus ambiguus (AMB) (Wehrwein et al., 2016). The telencephalon, including IC, ACC, and AMG, are involved in the high-level processing of visceral sensory information and initiate complete autonomic responses. The hypothalamus plays a central role in homeostasis and the integrated autonomic and endocrine responses required for adaptation to internal or external stimuli. Parasympathetic effects predominate when the anterior hypothalamus is stimulated, while sympathetic effects depend more on the posterior hypothalamus. The PAG forms the interface between the prosencephalon and the lower brainstem and plays a vital role in integrating autonomic and somatic responses to stress, pain regulation, and other adaptive functions. The dorsal motor nucleus of the vagus complex (DMV) and the nucleus tractus solitarius (NTS) make up the DVC. The NTS receives sensory afferents from the inferior vagus nerve, providing efferent signals to higher brain centers. The NTS neurons also integrate a large amount of sensory information with input from other brainstem and higher autonomic nuclei and transmit the information to the adjacent DMV, which contains preganglionic parasympathetic motor neurons that send output responses back to the viscera. Both the VLM and the AMB receive NTS projections, in which the AMB is located ventrolateral to the dorsal vagus nucleus and projects to the parasympathetic preganglionic of the heart. VLM contains neurons that control cardiovascular and respiratory function. The peripheral autonomic (parasympathetic and sympathetic) system consists of several functionally and anatomically independent neuronal pathways. Signals are generated by central integration and conducted *via* preganglionic neurons to paravertebral and prevertebral ganglia, and finally precisely to effector cells and produce autonomous responses in the cardiovascular system, respiratory system, gastrointestinal system, and some other functions.

## Mechanisms underlying autonomic nervous system mediation of acupuncture effects

Several retrospective studies have shown that the ANS is one of the key neural pathways responsible for the regulating effects of acupuncture at specific acupoints (Li et al., 2013). Here, we review the central and peripheral ANS pathways through which acupuncture regulates multiple systems (Table 1).

**TABLE 1:** Effects and autonomic regulatory mechanisms of acupuncture treating diseases.

References	Model	Acupoints	Intervention parameters	Effects	Neuron activity in autonomic brain regions	Autonomic nerve activity
Zhao et al., 2020	Visceral hypersensitivity rats	ST25, ST37	EA: 2/100 Hz, 2 mA, 20 min	Improves IBS visceral hypersensitivity	MT/ACC: Inhibition of astrocyte activity↓	/
Weng et al., 2015	Visceral hypersensitivity rats	ST25, ST37	EA: 2/100 Hz, 2 mA, 20 min	Reduce sensitivity to visceral pain	PFC/ACC: P2×3↓	/
Dhond et al., 2008	Healthy volunteers	PC6	MA: Manually twirled (± 180°) at 0.5 Hz	Enhance the post-stimulation spatial extent of resting brain networks	DMN connectivity with AMG/ACC/PAG/ hippocampal↑	SN↓, PSN↑, HRV(LFu↓,HFu↑)
Pang et al., 2021	Premenstrual syndrome patients	SP6	EA: 1 Hz, 2 mA, 6 min	Enhance the amygdala functional connectivity	AMG-ACC: FC↑	/
Zhang X. H. et al., 2021	Neuropathic pain rats	ST36, GB34	EA: 2 Hz, 0.5–1.5 mA, 30 min	Analgesia, pain-related emotion↓	AMG: TNFα/IL-1β/GFAP/dopamine system↓	/
Hsu et al., 2020	Fibromyalgia mouse model	ST36	EA: 2 Hz, 1 mA, 15 min	Analgesia	AMG/somatosensory cortex/thalamus: TRPV1-ERK↓	/
Zhou et al., 2007	BK-induced increase of BP transient reflex in cats	PC5, PC6	EA: 2 Hz, 1–4 mA, 30 min	Suppress pressor reflex	VLM: Glu↓	SN↓
Cui et al., 2018b	Acute myocardial ischemia rats	HT7, HT5	EA: 2 Hz, 1.1 mA, 30 min	Reduce myocardial ischemic injury	PVN: Neuronal discharge↓	SN↓
Cui et al., 2018a	Acute myocardial ischemia rats	HT7, HT5	EA: 2 Hz, 1 mA, 30 min	Reduce myocardial ischemic injury	Hippocampus/NTS: Neuronal dischargeu	Vagus↑
Zhang et al., 2022	The hypertensive rats	ST36, ST40	EA: 2/15 Hz, 4 mA, 30 min.	Antihypertensive and sympathetic suppression	PVN: NPY↑	SN↓
Tjen-A-Looi et al., 2016	BK-induced increase of BP transient reflex in cats	PC5, PC6	EA: 2–4 Hz, 2–4 mA, 30 min	Reduce cardiovascular excitatory response	Responses of cardiovascular barosensitive VLM neurons↓	SN↓
Guo and Longhurst, 2010	Healthy rats	PC5, PC6	EA: 2 Hz, 30 min	Attenuates sympatho-excitatory responses	ARC/vlPAG: VGLUT3↑	SN↓
Li et al., 2010	BK-induced increase of BP transient reflex in cats	PC5, PC7	EA: 2 Hz, 1–4 mA, 30 min	Suppresses elevated blood pressure	ARC/vlPAG: VGLUT3↑	SN↓
Tjen-A-Looi et al., 2006	BK-induced increase of BP transient reflex in cats	PC5, PC6	EA: 2–4 Hz, 4 mA, 30 min	Reduce cardiovascular excitatory response	vlPAG: Neuronal discharge↑ VLM: Neuronal discharge↓	SN↓
Tjen-A-Looi et al., 2013	Hypercapnic acidotic rats	PC5, PC6	EA: 2–4 Hz, 1–4 mA, 0.5 ms, 30 min	Alleviates cardiovascular depressor responses	VLM/cVLM/AMB: GABA↑	SN↓, vagus↑
Zhang et al., 2013	Stress-induced hypertensive rats	ST36	EA: 4/20 Hz, 4 mA, 0.5 ms, 30 min	Antihypertensive	VLM: Apelin↓	/
Li et al., 2001	BK-induced increase of BP transient reflex in cats	PC5, PC6	EA: 5 Hz, 10–20 V, 1–2 mA, 0.5 ms,	Suppress pressor reflex	VLM: m-opioid receptors↑δ-opioid receptors ↑	/
Tjen-A-Looi et al., 2003	BK-induced increase of BP transient reflex in cats	PC5, PC6	EA: 2H z, 1–4 mA, 0.5 ms, 30 min	Suppress pressor reflex	VLM: Neuronal excitability↓	SN↓
Moazzami et al., 2010	BK-induced increase of BP transient reflex in cats	PC5, PC6	EA: 2 Hz, 2–4 mA, 0.5 ms, 30 min	Suppress pressor reflex	NRP: 5-HT↑; VLM: 5-HT1A receptors ↑	SN↓
Moazzami et al., 2010	BK-induced increase of BP transient reflex in cats	PC5, PC6	EA: 2 Hz, 2–4 mA, 0.5 ms, 30 min	Suppress pressor reflex	NRP: 5-HT↑; VLM: 5-HT1A receptors ↑	/
Guo et al., 2012	Cat treated with colchicine	PC5, PC6	EA: 2 Hz, 1–4 mA, 0.5 ms, 30 min	Regulate cardiovascular function	AMB: Excitability of preganglionic parasympathetic neurons ↑	PSN↑
Chen et al., 2016	Myocardial Ischemia Rats	PC5, PC7	EA: 2 Hz/15 Hz, 0.5 mA, 30 min	Anti-myocardial Ischemic Effect	AMB: The number of c-fos positive neuron	Vagus↑
Wang et al., 2015	Gastric distention rats	RN12, BL21	EA: 20–100 Hz, 2 mA, 20 min	Regulate gastric motility.	PVN/DVC: Neuronal discharge/ gastrointestinal hormones and receptors↑	Vagus↑
Wang et al., 2013	Gastric distention rats	RN12, BL21	EA: 20/100 Hz, 2–2.5 mA.	Regulate gastric motility.	DVC: Gastrointestinal hormones↑	Vagus
Lu M. et al., 2019	Gastric distention rats	PC6	EA: 2/15 Hz, 2 mA, 2 min.	Promotes gastric motility	DMV: GABA neurotransmitter↓	PSN↑, Vagus↑
Gao et al., 2012	Gastric distention rats	ST36	EA: 4 Hz, 2–3 mA, 0.5 ms, 20 min	Promotes gastric motility	DMV: NMDAR↑	/
Wang et al., 2007	Healthy rats	ST36, ST37	EA: 50 Hz, 20 V, 30 min	Regulate gastric motility.	NTS/DMV: Neuronal discharge↓	Vagus↑
Tian et al., 2018	Motion sickness rats	PC6, ST36	MA: Sparrow pecking technique, 30 times/min, 30 min	DMV: The number of p-IRβ- and p-ERK1/2-positive cells and insulin levels↑	/	/
He et al., 2018	RWIS rats	ST36	EA: 2/100 Hz, 1 mA, 0.5 ms, 30 min	Ameliorate RWIS-induced gastric mucosal lesions	PVN/CNA: Number of CRH neurons↓	/
Wu et al., 2010	NMSS-induced visceral hyperalgesia rats	ST36	EA: 10 Hz, 0.18 ms, 20 min	Attenuates visceral hyperalgesia	Brainstem and spinal cord: 5-HT↓	/
Zhou et al., 2013	LPS-induced tight junction injury in mice	/	VNS: 1 Hz, 5 V, 2 ms	Attenuated the disruption of tight junction in intestinal epithelium	/	Vagus↑
Du et al., 2013	Hemorrhagic shock rats	ST36	EA: 2–100 Hz, 2 mA, 1.5 h	Improves gut barrier dysfunction	/	Vagus↑
Tatewaki et al., 2003	Rats implanted with strain sensors	ST36	MA: Twisting right and left once every second for 30 s	Regulate gastric motility.	/	/
Li et al., 2007	Spinalized rats; splanchnic denervation in rats	Li11, ST13, ST36, CV6, BL21, ST21	MA: The needle was rotated clockwise and anti-clockwise at 2 Hz for 30 s.	Regulate gastric motility.	/	Li11, ST13, ST36: SN↓, Vagus↑; CV6, BL21, ST21: SN↑, Vagus↓
Torres-Rosas et al., 2014	LPS-induced endotoxemia mice	ST36	EA: 10 Hz 4 V, 40 mA, 50 μs	Anti-inflammatory	/	Vagus↑
Liu S. et al., 2020	LPS-induced endotoxemia mice	ST25, ST36	EA: ST35: 10 Hz, 0.5 mA, 15 min; ST25: 10 Hz, 3 mA, 15 min	Anti-inflammatory	DMV: Excitability of neurons↑	Vagus↑ SN↑
Lim et al., 2016	Endotoxemia mouse	ST36	MA: Slow rotation every 5 min, 30 min; EA: 1 V, 1 Hz, 2 ms, 30 min	Anti-inflammatory	NTS/DMV: excitability of neurons↑	Vagus
Yang et al., 2021	Post-operative ileus mouse	ST36	EA: 10 Hz, 1 mA, 0.4 ms, 20 min,	Ameliorates intestinal inflammation	DMV: GABAA↓	Vagus↑
Torres-Rosas et al., 2014	Sepsis mice	ST36	EA: 10 Hz, 40 mA, 15 min	Anti-inflammatory	/	Vagus↑
Song et al., 2015	Rats with thermal injury	ST36	EA: 3 Hz, 3 V, 2 ms, 12 min × 8	Anti-inflammatory	/	Vagus↑
Chi et al., 2018	Ischemic stroke rats	GV20, GV14	EA: 2/15 Hz, 1 mA, 30 min	Neuroprotective effect	DMV: Neuronal excitability↑	Vagus↑
Kim et al., 2008	carrageenan-induced paw inflammation mice	ST36	EA: 1/120 Hz, 0.5 ms, 1–3 mA, 30 min	Anti-inflammatory	/	SN↑
Li et al., 2008	CFA-induced inflammation and hyperalgesia rats	GB30	EA: 10 Hz, 3 mA, 0.1 ms	Inhibition of inflammatory edema	PVN: CRH neurons excitability↑	/
Zhang M. et al., 2021	Surgical trauma after hepatectomy patients	ST36, SP6	EA: 2/15 Hz, 2 mA, 30 min	Normalized HPA axis dysfunction after surgical trauma	PVN: SCGN↓	/
An et al., 2007	CCK-induced acute pancreatitis	ST36	EA: 2/100 Hz, 3–5V, 10 min	Anti-inflammatory, protect the pancreas	PVN: ACTH↑	/
Noda et al., 2015	Patients with depression	PC4, LI10, SP9 and SP6	Press needle stimulation	Antidepressant effect	/	Vagus↑
Liu et al., 2018	Ischemic stroke patients	ST36	TEA: 25 HZ, 10 mA, 1 h	A preventive effect on stroke-induced constipation	/	Vagusen SN↑

↑, upregulation; ↓, downregulation; ACC, anterior cingulate cortex; AMG, amygdala; AMB, ambiguus; ARC, arcuate nucleus; BK, bradykinin; BP, arterial blood pressure; CCI, chronic constriction injury; CFA, complete Freund adjuvant; C-CPA, affective pain modeled by complete Freund’s adjuvant- (CFA-) evoked conditioned place aversion; CPDC, chronic pain and depression comorbidity model; CRS, chronic restraint stress; CCK, cholecystokinin octapeptide; CSNS, carotid sinus nerve stimulation; CNA, central nucleus of the amygdala; CRH, corticotropin-releasing hormone; cAMP, cyclic adenosine monophosphate; CREB, cAMP response element-binding protein; CRF, corticotropin-releasing factor; DMN, default mode network; DMV, dorsal motor nucleus of the vagus complex; DRN, Doral raphe nuclei; DVC, dorsal vagal complex; EA, electroacupuncture; ERK: extracellular regulated protein kinases; GLUT-3, glucose transporter 3; HR, heart rate; HRV, heart rate variability; HPA, hypothalamic-pituitary-adrenal; IC, insular cortex; IBD, inflammatory bowel disease; IBS, irritable bowel syndrome; LPS, lipopolysaccharide; NAc, nucleus accumbens; NPS, neuropeptide S; NPSR, neuropeptide S receptor; NRP: nucleus raphe pallidus; NPY, neuropeptide Y; NOS, nitric oxide synthase; NMDAR, N-methyl-D-aspartate receptors; NE, norepinephrine; Nic, nicotine; NMSS, neonatal maternal separation stress; NTS, nucleus tractus solitarius; MA, Manual acupuncture; MT: medial thalamus; METH, methamphetamine; mBDNF, mature brain-derived neurotropic factor; PKA, protein kinase A; PFA, paraformaldehyde; PFC, prefrontal cortex; pTrkB, phosphorylated tropomyosin receptor kinase B; PSN, parasympathetic nerve; PVN, paraventricular nucleus; RWIS, restraint water immersion stress; rsFC, resting state functional connectivity; SCGN, secretagogin; TNBS, 2,4,6-trinitrobenzene sulfonic acid; SN, sympathetic nerve; TVNS, transcutaneous vagus nerve stimulation; VNS, vagus nerve stimulation; vlPAG, ventrolateral periaqueductal gray; VLM, ventrolateral medulla; Lfu, a sympathetic related HRV metric; HFu, a parasympathetic related HRV metric.

### Analgesia

There is a strong connection between visceral pain processing and CAN. Many of these regions are pain regulatory circuits that regulate nociceptive processing and pain affect by projecting to the spinal cord and trigeminal ganglia. In turn, pain-related regions, including the IC, ACC, and AMG, through their connections to the hypothalamus and brainstem, finally generate autonomous responses through preganglionic and parasympathetic neurons (Asahina et al., 2003).

The autonomic nuclei in prosencephalon regions such as ACC and PFC can be involved in acupuncture to alleviate visceral pain. Mice with inflammatory bowel disease (IBD) demonstrated depression and visceral pain, along with elevated P2Y12 expression in the PFC. P2Y12 receptors in microglia induce the expression of interleukin 1β (IL-1β), a key mediator of depression in the brain. Electroacupuncture at *Dachangshu* (BL25) has been found to significantly downregulate P2Y12 expression in the PFC, attenuating microglial activation and thereby inhibiting IL-1β expression in the PFC, leading to reductions in both visceral pain and depressive symptoms in mice with IBD (Li et al., 2021). It was found that reactive astrocytes in the medial thalamic nucleus (MT) and ACC are involved in visceral hypersensitivity in IBS, which could be alleviated by electroacupuncture at ST25 and ST37 by reducing astrocyte activation in the MT and ACC (Zhao et al., 2020). P2 × 3 receptors play an essential role in visceral pain in IBS and electroacupuncture at ST37, and ST25 has been shown to reduce sensitivity to visceral pain by downregulating P2 × 3 receptor expression in the PFC and ACC of rats (Weng et al., 2015). This evidence shows that autonomic nuclei such as ACC and PFC can also be involved in acupuncture to alleviate visceral pain. However, further studies are needed to determine whether acupuncture exerts its analgesic effect by affecting peripheral autonomic nerves through these brain regions.

The AMG complex regulates emotions induced by sensory stimuli such as pain. Strengthening the connection between the AMG and the related nuclei in the PFC, hypothalamus, PAG, and VLM has been proposed to be the potential mechanism underlying the relief of pain-associated emotion by acupuncture, together with other therapeutic effects (Dhond et al., 2008; Liu J. et al., 2016). It was found that the pathogenesis of the premenstrual syndrome is closely related to the abnormal functional network of the AMG (Deng et al., 2018). Electroacupuncture at SP6 increased the functional connection (FC) between the AMG and the brainstem, the right hippocampus, the right orbitofrontal cortex, and the bilateral ACC. It decreased the FC between the AMG and the left thalamus and bilateral supplementary motor area (SMA), improving the symptoms of premenstrual syndrome (Pang et al., 2021). Electroacupuncture at ST36 and *Yanglingquan* (GB34) can upregulate dopamine (DA) synthesis and release in the AMG and increase the expression of both dopamine receptor D1 (DRD1) and dopamine receptor D2 (DRD2), thereby activating DRD1 and DRD2 signaling. This, in turn, inhibits astrocyte activation, reduces the expression of tumor necrosis factor α (TNF-α), IL-1β and glial fibrillary acidic protein in the AMG and suppresses excessive neuroinflammation, ultimately leading to pain relief and improved mood (Zhang X. H. et al., 2021). Electroacupuncture at ST36 has also been shown to reduce neuroinflammation by downregulating the TRPV1-PERK signaling pathway in the mouse AMG, somatosensory cortex, and thalamus to attenuate acid saline injection-induced nociceptive hyperalgesia, suggesting that pain-related signaling pathways in the AMG are involved in acupuncture analgesia relieving pain-related emotions (Hsu et al., 2020).

**FIGURE 1:**
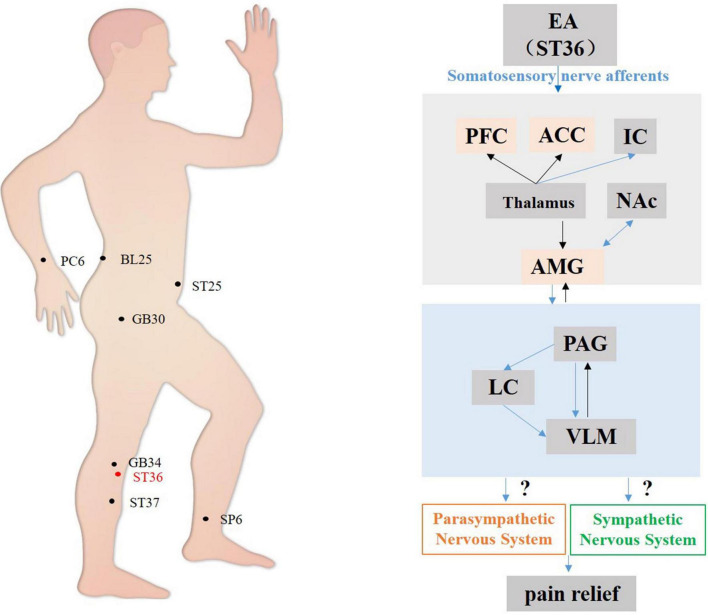
Hypothetical diagram of the central nervous circuits of autonomic nerves involved in acupuncture-mediated analgesia. Ascending pathway (black line); descending pathway (blue line). S1, the primary somatosensory cortex; S2, the second somatosensory area; PFC, prefrontal cortex; ACC, anterior cingulate cortex; NAc, nucleus accumbens; AMG, amygdala; PAG, periaqueductal gray; LC, locus coeruleus; VLM, ventrolateral medulla.

In summary, the mechanisms associated with acupuncture-mediated analgesia have been investigated relatively systematically. It has been found that the analgesic effects of acupuncture are primarily related to the regulation of the ascending activating system and descending inhibition system (Zhang et al., 2014). ST36 on the Stomach Channel of Foot-Yangming is the most commonly used and effective acupoint in acupuncture analgesia research. The limbic systems, such as the AMG, PFC, and ACC in the prosencephalon, are essential in the regulation of pain emotion. These autonomic cortical regions form a complex pain regulatory network with autonomic nuclei related to descending inhibition systems such as PAG, RVM, NTS, and dorsal reticular nucleus to participate in acupuncture analgesia and alleviate pain-related emotion (Figure 1). However, there are few reports on the effects of acupuncture on autonomic nerve indices during the regulation of these autonomic nerve nuclei, nor on how the central autonomic nerve network can influence the sympathetic and parasympathetic efferents to mediate the effects of acupuncture.

### Cardiovascular system

It has been found that electroacupuncture at the HT7-*Tongli* (HT5) acupoints can reduce myocardial ischemia by inhibiting the excitability of neurons projecting from the hippocampus to the paraventricular nucleus (PVN) and inhibiting sympathetic efferent nerves. This regulatory mechanism involves the hippocampal-PVN-sympathetic pathway, with the critical neurons in the PVN acting as interneurons (Cui et al., 2018a). Hypothalamic sympathetic efferents to the muscular, vascular bed are mainly responsible for regulating blood pressure. Electroacupuncture at ST36 and ST40 has been found to reduce chronic stress-induced sympathetic activity enhancement, and can upregulate the expression of neuropeptide-y (NPY) in hypothalamic PVN and plasma, and exerts a hypotensive effect in hypertensive rats, which was counteracted by PVN microinjection with NPY receptor antagonist (Zhang et al., 2022). Responses of cardiovascular barosensitive VLM neurons evoked by splanchnic nerve stimulation were reduced by electroacupuncture and subsequently restored by opioid receptor blockade in the PVN. Electroacupuncture at PC5–PC6 decreased splanchnic-evoked activity in cardiovascular barosensitive PVN neurons that also project directly to the VLM. PVN neurons projecting to the VLM were labeled with a retrograde tracer and were found to be co-localized with μ-opioid receptors and juxtaposed to endorphinergic fibers. Thus, the PVN and its projections to the VLM are essential in processing acupuncture treatment of elevated blood pressure through a PVN-opioid mechanism (Tjen-A-Looi et al., 2016).

The vagus nerve-NTS pathway plays an important role in cardiovascular reflex regulation. It has been found that the hippocampus-NTS-vagus pathway is involved in the cardioprotective effects of electroacupuncture in the heart meridian. By stimulating the hippocampus-NTS-vagus nerve pathway, electroacupuncture at HT7-HT5 can decrease HR, increase mean arterial pressure (MAP), and increase the HR-blood pressure product (RPP) in myocardial ischemia-susceptible rats (Cui et al., 2018b). When electroacupuncture stimulates HT7 and HT5, the NTS transmits the acupuncture signal to the hippocampus for integration of the signal, output, modulation of excitability in specific neurons in the NTS, and finally, the vagal signal from the NTS is transmitted to the heart where it regulates cardiac activity (Cui et al., 2018b). In addition, the increased pressor and depressor responses to microinjection of γ-aminobutyric acid (GABA) receptor agonists and antagonists into the NTS in hypertensive rats were found to be blunted by the electroacupuncture treatment. The electroacupuncture treatment also attenuated increased GABA receptor expression in the NTS of hypertensive rats. These results suggest that electroacupuncture at ST36 may positively affect renovascular hypertension *via* modulating functional GABA receptors in the NTS (Zhang Q. et al., 2018).

The PAG is a vital integration area for autonomic afferents and efferents. It was reported that after PC5–PC6 electroacupuncture stimulation, some neurons in the arcuate nucleus (ARC) and vlPAG of mice injected with tracer were reactive for c-Fos, retrograde tracer, and vesicular glutamate transporter 3 (VGLUT3), suggesting that electroacupuncture at PC5–PC6 attenuates sympathetic excitatory responses by activating the ARC-vlPAG-VLM (Guo and Longhurst, 2010). Specifically, in the BK-induced increase of the BP transient reflex in cats, excitatory glutamatergic connections between the ARC and vlPAG help to maintain the inhibitory effects of electroacupuncture at PC5–PC6 on the cardiovascular system (Li et al., 2010). It was also found that electroacupuncture at PC5–PC6 activates projections from the vlPAG to the VLM and inhibits the sympathetic excitatory cardiovascular response modulated by the premotor neurons of the VLM (Tjen-A-Looi et al., 2006).

The VLM contains neurons that control vasodilatory tone, cardiac function, and respiration. Electroacupuncture at PC5–PC6 can inhibit sympathetic efferents by activating GABA or opioid peptide neurons in the VLM, which, in turn, modulate visceral function (Tjen-A-Looi et al., 2013; Guo and Malik, 2019). Recent reports have described a new multifunctional peptide, apelin, closely related to blood pressure increases. Repeated electroacupuncture stimulation at ST36 can reduce the expression of both apelin and its receptor in the VLM of hypertensive rats (Zhang et al., 2013). Acupuncture at the PC5–PC6 acupoints attenuate the visceral sympathetic excitatory response by affecting μ and δ opioid receptors in the VLM (Li et al., 2001; Tjen-A-Looi et al., 2003). Electroacupuncture at PC5–PC6 can also inhibit the action of glutamate in the VLM through opioid receptors, thereby attenuating the bradykinin (BK)-induced visceral sympathetic excitatory reflex in cats (Zhou et al., 2007).

The nucleus raphes pallidus (NRP) is one of the important nuclei regulating the tension of sympathetic nerves. It was found that electroacupuncture at PC5–PC6 activated serotonin (5-hydroxytryptamine, 5-HT)-containing neurons in the NRP and suppressed the cardiovascular sympathetic excitatory reflex by activating 5-HT1a receptors in the VLM (Moazzami et al., 2010). The AMB contains 5-HT neurons that project to the spinal cord and regulate sympathetic nerve activity as well as cardiovascular function. It has been shown that electroacupuncture at the PC5–PC6 activates preganglionic parasympathetic neurons in the AMB and attenuates excitatory cardiovascular reflexes through the enkephalinergic mechanism (Guo et al., 2012). A recent study has shown that electroacupuncture at PC6–PC7 modulates the expression of choline acetyltransferase (ChAT), the vesicular acetylcholine transporter (VChAT), and c-Fos proteins in the AMB, as well as controlling the release of ChAT and parasympathetic nerve tension through activation of AMB neurons (Chen et al., 2016).

**FIGURE 2:**
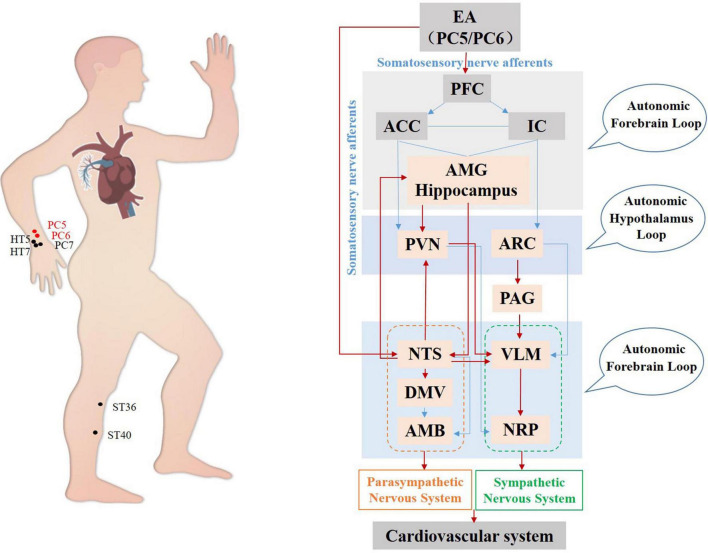
Hypothetical diagram of the central nervous circuits in which autonomic nerves contribute to regulating cardiovascular function by acupuncture. The red line shows autonomic neural pathways that have been demonstrated to influence the effects of acupuncture; the blue line shows known autonomic neurophysiological pathways that, although likely to mediate the effects of acupuncture, have not been confirmed. EA, electroacupuncture; PFC, prefrontal cortex; ACC, anterior cingulate cortex; IC, including the insular cortex; AMG, amygdala; PVN, paraventricular nucleus; ARC, arcuate nucleus; PAG, periaqueductal gray; NTS, nucleus tractus solitarius; DMV, dorsal motor nucleus of the vagus nerve; VLM, ventrolateral medulla; AMB, nucleus ambiguus; NRP, nucleus raphes pallidus.

In summary, the present review concentrated mainly on the influence of the midbrain and brain stem in acupuncture-mediated effects on the cardiovascular system, as shown in Figure 2. The acupoints on the Heart Channel of Hand-Shaoyin and the Pericardial Channel of Hand-Jueyin are commonly used for treating diseases of the cardiovascular system, and PC5 and PC6 are the most commonly used and effective acupoints for regulating cardiovascular function. Acupuncture activates the PVN hypothalamic nuclei and the PAG midbrain nuclei, thereby inhibiting the medulla oblongata VLM nucleus before activating sympathetic neurons and their subsequent reduction of cardiovascular excitation. Moreover, acupuncture stimulates centers associated with the vagus nerves, including the hypothalamic and midbrain nuclei, thereby regulating cardiovascular function (Tjen-A-Looi et al., 2013).

### Gastrointestinal system

The autonomic nuclei in the DVC and PVN play a leading role in regulating gastrointestinal function. Electroacupuncture at *Zhongwan* (RN12) and *Weishu* (BL21) can modulate gastrointestinal function *via* the hypothalamic PVN-DVC-vagus nerve-gastric nerve pathway, shown by electroacupuncture-mediated upregulation of motilin, gastrin, and their receptors in the hypothalamic PVN, as well as electroacupuncture enhancement of the firing of gastric dilatation-sensitive extracellular neurons in the DVC, thereby regulating gastric motility (Wang et al., 2015). It has been found that signals generated by electroacupuncture stimulation at BL21 and RN12 acupoints in rats were concentrated in the hypothalamic PVN and DVC and were involved in regulating gastric motility through the upregulation of gastrointestinal hormones in the gastric lumen (Wang et al., 2013). Electroacupuncture at PC6 promotes efferent vagal activity and increases gastric motility primarily by inhibiting GABA transmission in the DMV and reducing the inhibition of vagal efferent fibers (Lu M. et al., 2019). Moreover, there is evidence that neurons in the DMV and their signaling pathways regulate gastrointestinal function directly. For example, studies have found that NMDA receptor-dependent synaptic activity in the DMV of the vagus nerve mediates electroacupuncture stimulation at ST36 to enhance gastric motility (Gao et al., 2012; Browning and Carson, 2021); It has also been demonstrated that electroacupuncture at ST36 and ST37 can activate neurons within the NTS and DMV, thereby regulating gastric activity (Wang et al., 2007). Acupuncture at PC6 and ST36 can also significantly alleviate gastrointestinal symptoms caused by motion sickness (MS) through the IRβ-ERK1/2-dependent insulin receptor signaling pathway in the DMV (Tian et al., 2018). Electroacupuncture at BL21 and RN12 can enhance gastrointestinal function by activating NTS and DVC neurons and vagal efferents (Wang et al., 2015). In addition, it was found that restraint water-immersion stress (RWIs) induced significant activation of CRH neurons in the AMG and activated brainstem parasympathetic circuits that regulate gastric digestion through direct projections to neurons in the NTS and DMV, leading to gastric mucosal injury. In contrast, electroacupuncture at ST36 attenuated RWIs-induced gastric mucosal injury by inhibiting CRH neuronal activation in the AMG and PVN (He et al., 2018). Electroacupuncture at ST36 has also been suggested to reduce visceral hyperfunction in IBS by downregulating 5-HT neuronal activity in the brainstem and spinal cord (Wu et al., 2010).

Afferent signals induced by acupoint stimulation directly regulate visceral autonomic activity after spinal integration. Calcitonin gene-related peptide (CGRP) neurons in the posterior horn of the spinal cord are synaptically linked to GABA-ergic neurons in the posterior horn, and GABAergic neurons inhibit lateral horn sympathetic preganglionic neuronal excitability. Li et al. (2007) demonstrated that different regulatory effects of acupuncture on gastrointestinal function at the spinal cord level appear to be closely related to the direct control of sympathetic and parasympathetic pathways by the spinal cord. Manual acupuncture stimulation to the acupoints on forelimbs *Quchi* (Li11), upper chest-dorsum *Qihu* (ST13), and hindlimbs ST36, which are distant to the region of gastric innervation, produces a facilitative response of gastric motility with increased activities of the vagus and/or a slight inhibition in the activity of sympathetic nerves. Acupuncture stimulation to the acupoints on lower-chest *Qihai* (CV6), middle-dorsum BL21, and whole abdomen *Liangmen* (ST21), which are homo-segmental to the region of gastric innervation, induces an inhibitory response of gastric motility with increased activities of sympathetic nerves and/or a slight inhibition in the activity of vagal nerves, suggesting that the spinal cord can participate directly in gastrointestinal function by modulating both sympathetic and parasympathetic pathways (Tatewaki et al., 2003; Li et al., 2007).

In the peripheral autonomic system, significant differences in the effects of electroacupuncture at ST36 on gastric electrical conduction were observed before and after bilateral resection of the vagus, in electroacupuncture-mediated alterations in frequency before resection, and unchanged electrical activity after resection. This indirectly confirms the inability of acupuncture to regulate visceral function without mediating the visceral autonomic nerves (Wang et al., 2007). In a rat model of intestinal barrier dysfunction resulting from severe burns leading to intestinal ischemia, vagus nerve stimulation reduced intestinal epithelial permeability by modulating the vagus nerve and increasing the expression and correct localization of tight junction proteins (Zhou et al., 2013; Van Houten et al., 2015). Similarly, electroacupuncture at ST36 reduced intestinal permeability through vagus-mediated anti-inflammatory mechanisms, preventing intestinal barrier dysfunction after prolonged hemorrhagic shock (Du et al., 2013).

Acetylcholine and norepinephrine are synthesized and stored in presynaptic nerve endings. Initiating specific effects, they bind to specific ion channels or G-protein-coupled receptors in postsynaptic neurons, smooth muscle cells, or glandular epithelium cells upon release. Adrenaline binds to adrenergic receptors to inhibit gastric motility. At the same time, the excitatory fibers of parasympathetic nerves are generally cholinergic and play excitatory roles through the interaction of acetylcholine with muscarinic (M) and nicotinic (N) acetylcholine receptors. In contrast, inhibitory fibers interact with receptors on effector cells to temporarily increase the stomach’s volume. By observing the excretion of phenol red in mouse feces, we found that deletion of the M2/3 receptor significantly inhibited acupuncture-induced motility of the stomach, jejunum, and colon. In addition, knockout of the M1/2 receptor significantly reduced inhibition of gastric and jejunal motility induced by electroacupuncture at ST25 or ST37 while, compared with wild-type mice, significant reductions in gastric motility in the M2/3-knockout mice were observed. Acupuncture at the ST36 acupoint significantly increased gastrointestinal activity in M1/2 receptor knockout mice but reduced motility in M2/3 knockouts without altering gastrointestinal activity in wild-type mice (Gao et al., 2016; Lu M. J. et al., 2019). Acupuncture cannot control the activity of other organs, such as the heart, lungs, and so forth, without the involvement of autonomic nerves (Tjen-A-Looi et al., 2003; Zhang X. F. et al., 2018); nevertheless, the functions of particular neurotransmitters in these processes need to be clarified.

**FIGURE 3:**
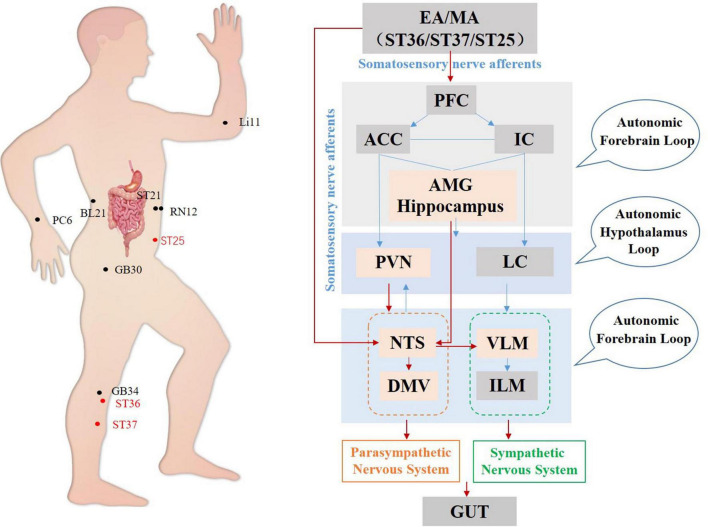
Hypothetical diagram of central nervous circuits in which autonomic nerves participate in acupuncture-mediated regulation of the gastrointestinal system. The red line shows autonomic neural pathways that have been demonstrated to influence the effects of acupuncture; the blue line shows known autonomic neurophysiological pathways that, although likely to mediate the effects of acupuncture, have not been confirmed. PFC, prefrontal cortex; ACC, anterior cingulate cortex; IC, including the insular cortex; AMG, amygdala; PVN, paraventricular nucleus; LC, locus coeruleus; NTS, nucleus tractus solitarius; DMV, dorsal motor nucleus of the vagus nerve; VLM, ventrolateral medulla; ILM, intermediolateralis nucleus.

In summary, regarding the gastrointestinal system, current studies have focused mainly on the regulation of the brainstem by acupuncture. The complex network formed by central autonomous nuclei such as the hypothalamus, DVC, NTS, and VLM affects sympathetic and parasympathetic efferents mediating acupuncture gastrointestinal function (Figure 3). The acupoints on the Stomach Channel of Foot-Yangming and the Large Intestine Channel of Hand-Yangming are most often used, while ST36, ST37, and ST25 are the most commonly used and effective acupoints in acupuncture regulating gastrointestinal function. Electroacupuncture at these acupoints can inhibit the activity of GABAergic neurons in DMV, weaken its inhibitory effect on the vagus nerve, and improve gastrointestinal motility in rats (Lu M. et al., 2019; Yang et al., 2021).

### Anti-inflammatory action

Studies have shown that the DVC, particularly the DMV, is important in regulating vagal anti-inflammatory effects. For example, studies have found that manual acupuncture at ST36 activates neurons and glial cells expressing glutamate and purinergic receptors in the DVC, enhances vagal activity activating splanchnic nerves by increasing synaptic transmission in the DVC circuit, induces an anti-inflammatory response in splenic macrophages, and reduces TNF-α levels in both serum and splenic tissue (Lim et al., 2016). Electroacupuncture of ST36 inhibits the expression of ChAT^+^ neuronal GABA receptors in the DMV. Its excitation of the vagus nerve attenuates post-operative intestinal inflammatory responses by activating the α7 nicotinic acetylcholine receptor (α7-nAChR)-mediated Janus kinase 2 (JAK2)/signal transducer and activator of transcription 3 (STAT3) signaling pathway in macrophages (Yang et al., 2021).

The ANS can regulate acute and chronic inflammatory responses at local and systemic levels (Ulloa, 2005). Neurotransmitters released from sympathetic and parasympathetic nerve endings bind to their respective receptors located on the surface of immune cells to initiate immunomodulatory responses. Autonomic nerves can also influence the number of β-adrenergic receptors expressed on immune cells through activation of the HPA axis and the subsequent release of cortisol (Nakada et al., 1987). In addition, activation of β2-adrenergic receptors enhances the glucocorticoid receptor gene expression rate by activating the downstream phosphatidylinositol 3-kinase (PI3K) pathway, leading to a controlled and enhanced immune response to disease (Schmidt et al., 2001). Typically, in acute inflammatory states, the HPA axis and SNS act synergistically or are coupled to each other, leading to enhanced release of cortisol and SNS neurotransmitters, which contribute to the regulation of immune cell activity (Borovikova et al., 2000; Straub, 2004). Studies have shown that electroacupuncture at *Huantiao* (GB30) activates corticotropin-releasing hormone (CRH) neurons in the PVN of the hypothalamus and the HPA axis, thereby reducing the inflammatory response (Li et al., 2008). Electroacupuncture at ST36 and SP6 also reduces HPA axis hyperactivity resulting from surgical trauma after hepatectomy by decreasing the transcription and synthesis of PVN secretagogin (SCGN) and inhibiting the synthesis and release of CRH (Zhang M. et al., 2021). In the cholecystokinin (CCK)-induced acute pancreatitis model, electroacupuncture at ST36 stimulated the hypothalamic or dorsal vagal network, leading to the release of the melanocortin adreno-cortico-tropic-hormone (ACTH), thereby inhibiting NF-κB activity and pro-inflammatory cytokine production, reducing inflammation and having a protective effect on the pancreas (An et al., 2007).

Various animal models have demonstrated the anti-inflammatory and analgesic effects of vagus-mediated acupuncture. ST36 electroacupuncture improved the survival rate of mice with bacterial peritonitis by preventing ischemic and hemorrhagic intestinal damage and reducing the serum levels of TNF, MCP1, IL-6, and IFN-γ; these effects were blocked by vagotomy (Torres-Rosas et al., 2014). Studies have shown that electroacupuncture at ST36 can activate the production of adrenal dopa decarboxylase, leading to the release of dopamine throughout the body, thereby inhibiting the release of inflammatory factors by macrophages (Torres-Rosas et al., 2014). It has also been reported that auricular electrical stimulation, acupuncture, and electroacupuncture at ST36 reduce serum concentrations of the inflammatory cytokines TNF-, IL-1, and IL-6 and improve renal and lung injury in sepsis-affected rodents. Electroacupuncture at ST36 has also been found to prevent inflammation and lung tissue damage caused by burns through regulating the activity of the vagus nerve (Song et al., 2015). Electroacupuncture at *Baihui* (GV20) and *Dazhui* (GV14) has been found to improve cerebral blood perfusion in mouse models of ischemic stroke, reducing brain injury, apoptosis, oxidative stress, and inflammation through vagal stimulation (Chi et al., 2018).

The SNS runs through the spinal cord and innervates most internal organs, and preganglionic sympathetic nerves can activate photochromogenic cells in the adrenal gland to release catecholamines into the blood (Han et al., 2003; Ulloa et al., 2017). Electroacupuncture can induce local and systemic catecholamine secretion by stimulating sympathetic nerves, with specific effects closely related to the frequency. Studies have shown that high-frequency electroacupuncture stimulation at ST36 can inhibit carrageenan-induced inflammation through preganglionic innervation of the adrenal gland (Kim et al., 2008). In contrast, low-frequency electroacupuncture stimulation at ST36 reduced inflammation in a mouse air pouch inflammation model, surgical trauma, sepsis, and arthritis through local sympathetic post-ganglion nerves (Wang et al., 2005; Kim et al., 2006; Santos-Almeida et al., 2017). Therefore, high-frequency electroacupuncture appears to activate the preganglionic nerves of the adrenal medulla to induce systemic catecholamine release. In contrast, electroacupuncture with a low frequency may stimulate specific sympathetic post-ganglionic nerves to influence the local release of neurogenic norepinephrine.

**FIGURE 4:**
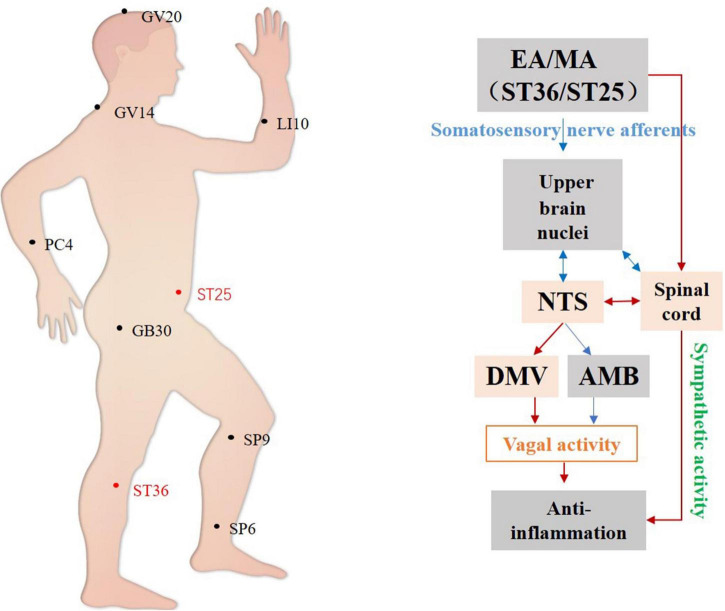
Hypothetical diagram of the central nervous circuits of autonomic nerves involved in acupuncture-mediated anti-inflammatory effects. The red line shows autonomic neural pathways that have been demonstrated to influence the effects of acupuncture; the blue line shows known autonomic neurophysiological pathways that, although likely to mediate the effects of acupuncture, have not been confirmed. NTS, nucleus tractus solitarius; DMV, dorsal motor nucleus of the vagus nerve; AMB, nucleus ambiguus.

To summarize, studies on the anti-inflammatory effects of acupuncture have focused mainly on the brainstem (Figure 4) and ST36 and ST25 are the most commonly used and effective acupoints. It has been demonstrated that manual acupuncture at ST36 can activate vagus nuclei in the NTS, DMV, and AMB brain regions, leading to an anti-inflammatory action while high-intensity electroacupuncture at ST25 activates the spinal-sympathetic pathways, producing anti-inflammatory effects (Straub, 2004; Lim et al., 2016).

## Discussion and conclusion

Based on the underlying functional pathways and physiological roles of the ANS, we speculate that the ANS plays a significant role in the central nervous circuit activated by acupuncture, leading to the modulation of cardiovascular and gastrointestinal functions, as well as anti-inflammatory and analgesic functions (Salman, 2016; Park and Namgung, 2018; Yang and Chang, 2019; Bonaz et al., 2021). It is worth noting that the prosencephalon is a higher autonomic nucleus that senses pressure, integrates information, and can regulate cardiovascular, gastrointestinal, and other visceral functions. However, further investigation is needed into whether acupuncture can regulate the cardiovascular and gastrointestinal systems by regulating the prosencephalon autonomic nuclei. However, the potential mechanisms by which acupuncture regulates other visceral functions in the lungs, liver, and kidney are not known, which represents a limitation of the present study, and which requires further study and analysis.

Overall, we speculate that acupuncture may excite or inhibit autonomic nuclei within the prosencephalon (IC, PFC, ACC, AMG, and hippocampus), hypothalamus, PVN, PAG, NTS, DMV, VLM, and AMB, then regulating vagal, sympathetic, and parasympathetic efferents to play acupuncture effect. Different autonomic nuclei are involved in regulating the function of organs in diverse systems. For instance, acupuncture treats cardiovascular disease by activating the hypothalamic PVN nuclei and the PAG midbrain nuclei, together with inhibition of medulla oblongata VLM nucleus, and finally, inhibition of cardiac sympathetic nerve excitability. Furthermore, acupuncture activates the vagus center and efferent vagus nerve to regulate cardiovascular function (Tjen-A-Looi et al., 2013). A complex network of central autonomic nuclei, including the hypothalamus, DMV, NTS, and VLM, collaborates with the peripheral ENS to mediate the effect of acupuncture on gastrointestinal function. The PFC, IC, and ACC are additional cortical autonomic nuclei implicated in pain regulation. The PAG, RVM, NTS, AMG, and dorsal reticular nucleus are autonomic nuclei connected to the pain descending inhibitory system and play a role in the complex network underlying acupuncture analgia. Current knowledge indicates that the central autonomic nuclei are implicated in acupuncture modulation of the gastrointestinal and cardiovascular systems. The inflammatory response is mainly located in the midbrain and brain stem. In addition, there have been no systematic investigations into how the central ANS mediates acupuncture analgesia and drug addiction. Most current studies focus on the activity and related molecular changes of autonomic nuclei induced by acupuncture but there is a dearth of studies on how neural circuits regulate sympathetic and parasympathetic efferents.

In the periphery, acupuncture can improve organ function, control inflammation, and exert analgesic effects through the modulation of autonomic nerves. For example, electroacupuncture at ST36 and PC6 can improve gastrointestinal motility in rats with gastric dilatation by stimulating vagus nerves (Wang et al., 2007; Lu M. et al., 2019); and electroacupuncture at PC5 and PC6 can reduce cardiovascular excitatory response and regulate blood pressure by the stimulating the vagus nerve and inhibiting sympathetic nerves (Chen et al., 2016). However, most of the current studies have used the loss-and-gain method to investigate the contribution of efferent sympathetic and parasympathetic nerves to the effects of acupuncture, and have been limited to the assessment of the anti-inflammatory effects of acupuncture on ANS-mediated functions, and further investigation is needed into the afferent nerves, central integration, and efferent autonomic nerves mediating other effects of acupuncture.

Recent research indicates that the involvement of the ANS in the anti-inflammatory effects of acupuncture depends on the acupoint’s selection and intensity. For example, low-intensity (0.5 mA) electroacupuncture at the hindlimb acupoint ST36, but not the abdominal ST25, inhibited LPS-induced systemic inflammation by activating efferent vagus. High-intensity (3.0 mA) electroacupuncture ST25 could activate NPY^+^ sympathetic nerves, which project to the spleen to inhibit LPS-induced inflammation, while low-intensity (0.5 mA) stimulation of ST25 could not activate this pathway. Before LPS-induced systemic inflammation, electroacupuncture ST25 could exert an anti-inflammatory effect by activating peripheral NPY^+^ sympathetic neurons. Still, after LPS-induced inflammation, the same abdominal acupoints and stimulation intensity showed pro-inflammatory effects (Liu S. et al., 2020). Recently, Liu et al. (2021) have shown that PROKR2Cre-marked sensory neurons, which innervate the deep hindlimb fascia (for example, the periosteum) but not abdominal fascia (for example, the peritoneum), are crucial for driving the vagal-adrenal axis, provide a neuroanatomical basis for the selectivity and specificity of acupoints in driving specific autonomic pathways.

Similarly, in terms of the mechanism of acupuncture analgesia and regulation of visceral function, the autonomic nerve pathway that mediates the acupuncture effect caused by different acupoints and parameters may have specific rules that require more investigation. Furthermore, various acupuncture methods could result in different outcomes. Most studies on the effects of acupuncture have been included in the present review, and few have used manual acupuncture. Thus, additional research is required on whether and how various acupuncture methods control the ANS.

In conclusion, acupuncture controls multiple physiological responses by regulating the ANS. Both experimental and clinical results warrant future studies to decode the specific anatomical networks and molecular mechanisms connecting the acupuncture methods, acupoints, and the regulation of organ function both in healthy and disease state-dependent manner. The regulation of the ANS with acupuncture is a key entry point to explain the basic properties of acupuncture, such as acupoint specificity, and the scientific basis of meridians. Furthermore, these anatomical, molecular, and mechanistic studies will have major clinical implications to improve treatment efficacy as well as to determine the comorbidities that may limit acupuncture efficacy.

## Author contributions

Y-WL and WL: concept design, data collection, and manuscript writing. S-TW, Y-NG, and B-MD: preparation of the figures and table. Z-XL, S-JW, and LU: language modification and review of the manuscript text. YG and Z-FX: concept design and manuscript review. All authors contributed to the article and approved the submitted version.

## References

[B1] An H. J. Lee J. H. Lee H. J. Yang W. M. Park S. K. Hong S. H. (2007). Electroacupuncture protects against CCK-induced acute pancreatitis in rats. *Neuroimmunomodulation* 14 112–118. 10.1159/000107793 17804915

[B2] Asahina M. Suzuki A. Mori M. Kanesaka T. Hattori T. (2003). Emotional sweating response in a patient with bilateral amygdala damage. *Int. J. Psychophysiol.* 47 87–93. 10.1016/S0167-8760(02)00123-X 12543449

[B3] Bonaz B. Sinniger V. Pellissier S. (2021). Therapeutic potential of vagus nerve stimulation for inflammatory bowel diseases. *Front. Neurosci.* 15:650971. 10.3389/Fnins.2021.650971 33828455 PMC8019822

[B4] Borovikova L. V. Ivanova S. Zhang M. Yang H. Botchkina G. I. Watkins L. R. (2000). Vagus nerve stimulation attenuates the systemic inflammatory response to endotoxin. *Nature* 405 458–462. 10.1038/35013070 10839541

[B5] Browning K. N. Carson K. E. (2021). Central neurocircuits regulating food intake in response to gut inputs-preclinical evidence. *Nutrients* 13:908. 10.3390/Nu13030908 33799575 PMC7998662

[B6] Chen W. Chen S. P. Li C. W. Wang J. Y. Duanmu C. L. Chang X. R. (2016). [Does the nucleus ambiguus-vague nerve mediate anti-myocardial ischemic effect of electroacupuncture of “Neiguan” (PC 6)-”Jianshi” (PC 7) in myocardial ischemia rats]. *Zhen Ci Yan Jiu* 41 189–196. 29071904

[B7] Chi L. Du K. Liu D. Bo Y. Li W. (2018). Electroacupuncture brain protection during ischemic stroke: A role for the parasympathetic nervous system. *J. Cereb. Blood Flow Metab.* 38 479–491. 10.1177/0271678x17697988 28281385 PMC5851138

[B8] Cui S. Zhou Y. Wu S. Cao J. Zhu G. Zhou M. (2018a). Electroacupuncture improved the function of myocardial ischemia involved in the hippocampus-paraventricular nucleus-sympathetic nerve pathway. *Evid. Based Complement Alternat. Med.* 2018:2870676. 10.1155/2018/2870676 29507590 PMC5817851

[B9] Cui S. Wang K. Wu S. B. Zhu G. Q. Cao J. Zhou Y. P. (2018b). Electroacupuncture modulates the activity of the hippocampus-nucleus tractus solitarius-vagus nerve pathway to reduce myocardial ischemic injury. *Neural. Regen. Res.* 13 1609–1618. 10.4103/1673-5374.237124 30127122 PMC6126117

[B10] De Winter B. Y. Deiteren A. De Man J. G. (2016). Novel nervous system mechanisms in visceral pain. *Neurogastroenterol. Motil.* 28 309–315. 10.1111/Nmo.12785 26891060

[B11] Deng D. Pang Y. Duan G. Liu H. Liao H. Liu P. (2018). Larger volume and different functional connectivity of the amygdala in women with premenstrual syndrome. *Eur. Radiol.* 28 1900–1908. 10.1007/S00330-017-5206-0 29260367

[B12] Dhond R. P. Yeh C. Park K. Kettner N. Napadow V. (2008). Acupuncture modulates resting state connectivity in default and sensorimotor brain networks. *Pain* 136 407–418. 10.1016/J.Pain.2008.01.011 18337009 PMC2440647

[B13] Du M. H. Luo H. M. Hu S. Lv Y. Lin Z. L. Ma L. (2013). Electroacupuncture improves gut barrier dysfunction in prolonged hemorrhagic shock rats through vagus anti-inflammatory mechanism. *World J. Gastroenterol.* 19 5988–5999. 10.3748/Wjg.V19.I36.5988 24106399 PMC3785620

[B14] El-Badawy M. A. El Mikkawy D. M. (2016). Sympathetic dysfunction in patients with chronic low back pain and failed back surgery syndrome. *Clin. J. Pain* 32 226–231. 10.1097/Ajp.0000000000000250 25968450

[B15] Gao X. Qiao Y. Jia B. Jing X. Cheng B. Wen L. (2012). Nmda receptor-dependent synaptic activity in dorsal motor nucleus of vagus mediates the enhancement of gastric motility by stimulating ST36. *Evid. Based Complement Alternat. Med.* 2012:438460. 10.1155/2012/438460 23118791 PMC3478787

[B16] Gao X. Zhao Y. Su Y. Liu K. Yu X. Cui C. (2016). B 1/2 or M2/3 receptors are required for different gastrointestinal motility responses induced by acupuncture at heterotopic or homotopic acupoints. *PLoS One* 11:E0168200. 10.1371/Journal.Pone.0168200 27978539 PMC5158317

[B17] Guo Z. L. Li M. Longhurst J. C. (2012). Nucleus ambiguus cholinergic neurons activated by acupuncture: Relation to enkephalin. *Brain Res.* 1442 25–35. 10.1016/J.Brainres.2012.01.006 22306033 PMC3288561

[B18] Guo Z. L. Longhurst J. C. (2010). Activation of reciprocal pathways between arcuate nucleus and ventrolateral periaqueductal gray during electroacupuncture: Involvement of VGLUT3. *Brain Res.* 1360 77–88. 10.1016/J.Brainres.2010.08.102 20836994 PMC2962589

[B19] Guo Z. L. Malik S. (2019). Acupuncture activates a direct pathway from the nucleus tractus solitarii to the rostral ventrolateral medulla. *Brain Res.* 1708 69–77. 10.1016/J.Brainres.2018.12.009 30529283 PMC6378112

[B20] Hallman D. M. Lindberg L. G. Arnetz B. B. Lyskov E. (2011). Effects of static contraction and cold stimulation on cardiovascular autonomic indices, trapezius blood flow and muscle activity in chronic neck-shoulder pain. *Eur. J. Appl. Physiol.* 111 1725–1735. 10.1007/S00421-010-1813-Z 21221987

[B21] Han J. B. Oh S. D. Lee K. S. Choi K. S. Cho Y. W. Ahn H. (2003). The role of the sympathetic nervous system in moxibustion-induced immunomodulation in rats. *J. Neuroimmunol.* 140 159–162. 10.1016/S0165-5728(03)00211-X 12864984

[B22] He F. Wang M. Geng X. Ai H. (2018). Effect of electroacupuncture on the activity of corticotrophin-releasing hormone neurons in the hypothalamus and amygdala in rats exposed to restraint water-immersion stress. *Acupunct. Med.* 36 394–400. 10.1136/Acupmed-2017-011450 30173142

[B23] Ho R. Chung V. Wong C. Wu J. Wong S. Wu I. (2017). Acupuncture and related therapies used as add-on or alternative to prokinetics for functional dyspepsia: Overview of systematic reviews and network meta-analysis. *Sci. Rep.* 7:10320. 10.1038/S41598-017-09856-0 28871092 PMC5583250

[B24] Hsu H. C. Hsieh C. L. Lee K. T. Lin Y. W. (2020). Electroacupuncture reduces fibromyalgia pain by downregulating the TRPV1-pERK signalling pathway in the mouse brain. *Acupunct. Med.* 38 101–108. 10.1136/Acupmed-2017-011395 31941349

[B25] Kemp A. H. Quintana D. S. Gray M. A. Felmingham K. L. Brown K. Gatt J. M. (2010). Impact of depression and antidepressant treatment on heart rate variability: A review and meta-analysis. *Biol. Psychiatry* 67 1067–1074. 10.1016/J.Biopsych.2009.12.012 20138254

[B26] Kim H. W. Roh D. H. Yoon S. Y. Kang S. Y. Kwon Y. B. Han H. J. (2006). The anti-inflammatory effects of low- and high-frequency electroacupuncture are mediated by peripheral opioids in a mouse air pouch inflammation model. *J. Altern. Complement Med.* 12 39–44. 10.1089/Acm.2006.12.39 16494567

[B27] Kim H. W. Uh D. K. Yoon S. Y. Roh D. H. Kwon Y. B. Han H. J. (2008). Low-frequency electroacupuncture suppresses carrageenan-induced paw inflammation in mice via sympathetic post-ganglionic neurons, while high-frequency EA suppression is mediated by the sympathoadrenal medullary axis. *Brain Res. Bull.* 75 698–705. 10.1016/J.Brainresbull.2007.11.015 18355649

[B28] Koenig J. Loerbroks A. Jarczok M. N. Fischer J. E. Thayer J. F. (2016). Chronic pain and heart rate variability in a cross-sectional occupational sample: Evidence for impaired vagal control. *Clin. J. Pain* 32, 218–225. 10.1097/AJP.0000000000000242 25924095

[B29] Li A. Lao L. Wang Y. Xin J. Ren K. Berman B. M. (2008). Electroacupuncture activates corticotrophin-releasing hormone-containing neurons in the paraventricular nucleus of the hypothalammus to alleviate edema in a rat model of inflammation. *BMC Complement Altern. Med.* 8:20. 10.1186/1472-6882-8-20 18474100 PMC2408560

[B30] Li P. Tjen-A-Looi S. Longhurst J. C. (2001). Rostral ventrolateral medullary opioid receptor subtypes in the inhibitory effect of electroacupuncture on reflex autonomic response in cats. *Auton. Neurosci.* 89 38–47. 10.1016/S1566-0702(01)00247-8 11474645

[B31] Li P. Tjen-A-Looi S. C. Guo Z. L. Longhurst J. C. (2010). An arcuate-ventrolateral periaqueductal gray reciprocal circuit participates in electroacupuncture cardiovascular inhibition. *Auton Neurosci.* 158 13–23. 10.1016/J.Autneu.2010.05.006 20580325 PMC2976778

[B32] Li Q. Q. Shi G. X. Xu Q. Wang J. Liu C. Z. Wang L. P. (2013). Acupuncture effect and central autonomic regulation. *Evid. Based Complement Alternat. Med.* 2013:267959. 10.1155/2013/267959 23762116 PMC3677642

[B33] Li Y. Zhang H. Yang J. Zhan M. Hu X. Liu Y. (2021). P2Y12 receptor as a new target for electroacupuncture relieving comorbidity of visceral pain and depression of inflammatory bowel disease. *Chin Med.* 16:139. 10.1186/S13020-021-00553-9 34930362 PMC8686637

[B34] Li Y. Q. Zhu B. Rong P. J. Ben H. Li Y. H. (2007). Neural mechanism of acupuncture-modulated gastric motility. *World J. Gastroenterol.* 13 709–716. 10.3748/Wjg.V13.I5.709 17278193 PMC4066003

[B35] Lim H. D. Kim M. H. Lee C. Y. Namgung U. (2016). Anti-inflammatory effects of acupuncture stimulation via the vagus nerve. *PLoS One* 11:E0151882. 10.1371/Journal.Pone.0151882 26991319 PMC4798687

[B36] Liu C. H. Yang M. H. Zhang G. Z. Wang X. X. Li B. Li M. (2020). Neural networks and the anti-inflammatory effect of transcutaneous auricular vagus nerve stimulation in depression. *J. Neuroinflammation* 17:54. 10.1186/S12974-020-01732-5 32050990 PMC7017619

[B37] Liu J. Fang J. Wang Z. Rong P. Hong Y. Fan Y. (2016). Transcutaneous vagus nerve stimulation modulates amygdala functional connectivity in patients with depression. *J. Affect. Disord.* 205 319–326. 10.1016/J.Jad.2016.08.003 27559632

[B38] Liu S. Wang Z. Su Y. Qi L. Yang W. Fu M. (2021). A neuroanatomical basis for electroacupuncture to drive the vagal-adrenal axis. *Nature* 598 641–645. 10.1038/S41586-021-04001-4 34646018 PMC9178665

[B39] Liu S. Wang Z. F. Su Y. S. Ray R. S. Jing X. H. Wang Y. Q. (2020). Somatotopic organization and intensity dependence in driving distinct npy-expressing sympathetic pathways by electroacupuncture. *Neuron* 108 436.e7–450.e7. 10.1016/J.Neuron.2020.07.015 32791039 PMC7666081

[B40] Liu Z. Ge Y. Xu F. Xu Y. Liu Y. Xia F. (2018). Preventive effects of transcutaneous electrical acustimulation on ischemic stroke-induced constipation mediated via the autonomic pathway. *Am. J. Physiol. Gastrointest. Liver Physiol.* 315 G293–G293. 10.1152/Ajpgi.00049.2018 29746169

[B41] Liu Z. Yan S. Wu J. He L. Li N. Dong G. (2016). Acupuncture for chronic severe functional constipation: A randomized trial. *Ann. Intern. Med.* 165 761–769. 10.7326/M15-3118 27618593

[B42] Longhurst J. C. (2010). Defining meridians: A modern basis of understanding. *J. Acupunct. Meridian Stud.* 3 67–74. 10.1016/S2005-2901(10)60014-3 20633518

[B43] Lu L. Zhang Y. Tang X. Ge S. Wen H. Zeng J. (2022). Evidence on acupuncture therapies is underused in clinical practice and health policy. *Bmj* 376:E067475. 10.1136/Bmj-2021-067475 35217525 PMC8868048

[B44] Lu M. Chen C. Li W. Yu Z. Xu B. (2019). EA at PC6 promotes gastric motility: Role of brainstem vagovagal neurocircuits. *Evid. Based Complement Alternat. Med.* 2019:7457485. 10.1155/2019/7457485 31379967 PMC6662446

[B45] Lu M. J. Yu Z. He Y. Yin Y. Xu B. (2019). Electroacupuncture at ST36 modulates gastric motility via vagovagal and sympathetic reflexes in rats. *World J. Gastroenterol.* 25 2315–2326. 10.3748/Wjg.V25.I19.2315 31148903 PMC6529886

[B46] Manabe N. Tanaka T. Hata J. Kusunoki H. Haruma K. (2009). Pathophysiology underlying irritable bowel syndrome–from the viewpoint of dysfunction of autonomic nervous system activity. *J. Smooth Muscle Res.* 45 15–23. 10.1540/Jsmr.45.15 19377269

[B47] Moazzami A. Tjen-A-Looi S. C. Guo Z. L. Longhurst J. C. (2010). Serotonergic projection from nucleus raphe pallidus to rostral ventrolateral medulla modulates cardiovascular reflex responses during acupuncture. *J. Appl. Physiol.* 108 1336–1346. 10.1152/Japplphysiol.00477.2009 20133441 PMC2867542

[B48] Nakada M. T. Stadel J. M. Poksay K. S. Crooke S. T. (1987). Glucocorticoid regulation of beta-adrenergic receptors in 3T3-L1 preadipocytes. *Mol. Pharmacol.* 31 377–384. 3033466

[B49] Nance D. M. Sanders V. M. (2007). Autonomic innervation and regulation of the immune system (1987-2007). *Brain Behav. Immun.* 21 736–745. 10.1016/J.Bbi.2007.03.008 17467231 PMC1986730

[B50] Noda Y. Izuno T. Tsuchiya Y. Hayasaka S. Matsumoto K. Murakami H. (2015). Acupuncture-induced changes of vagal function in patients with depression: A preliminary sham-controlled study with press needles. *Complement Ther. Clin. Pract.* 21 193–200. 10.1016/J.Ctcp.2015.07.002 26256139

[B51] Pang Y. Liao H. Duan G. Feng Z. Liu H. Zou Z. (2021). Regulated aberrant amygdala functional connectivity in premenstrual syndrome via electro-acupuncture stimulation at sanyinjiao acupoint (Sp6). *Gynecol. Endocrinol.* 37 315–319. 10.1080/09513590.2020.1855633 33307896

[B52] Park J. Y. Namgung U. (2018). Electroacupuncture therapy in inflammation regulation: Current perspectives. *J. Inflamm. Res.* 11 227–237. 10.2147/Jir.S141198 29844696 PMC5963483

[B53] Rottenberg J. (2007). Cardiac vagal control in depression: A critical analysis. *Biol. Psychol.* 74 200–211. 10.1016/J.Biopsycho.2005.08.010 17045728

[B54] Salman I. M. (2016). Major autonomic neuroregulatory pathways underlying short- and long-term control of cardiovascular function. *Curr. Hypertens. Rep.* 18:18. 10.1007/S11906-016-0625-X 26838031

[B55] Santos-Almeida F. M. Domingos-Souza G. Meschiari C. A. Fávaro L. C. Becari C. Castania J. A. (2017). Carotid sinus nerve electrical stimulation in conscious rats attenuates systemic inflammation via chemoreceptor activation. *Sci. Rep.* 7:6265. 10.1038/S41598-017-06703-0 28740186 PMC5524712

[B56] Schmidt P. Holsboer F. Spengler D. (2001). Beta(2)-adrenergic receptors potentiate glucocorticoid receptor transactivation via G protein beta gamma-subunits and the phosphoinositide 3-kinase pathway. *Mol. Endocrinol.* 15 553–564. 10.1210/Mend.15.4.0613 11266507

[B57] Shankar N. Thakur M. Tandon O. P. Saxena A. K. Arora S. Bhattacharya N. (2011). Autonomic status and pain profile in patients of chronic low back pain and following electro acupuncture therapy: A randomized control trial. *Indian J. Physiol. Pharmacol.* 55 25–36. 22315807

[B58] Song X. M. Wu X. J. Li J. G. Le L. L. Liang H. Xu Y. (2015). The effect of electroacupuncture at ST36 on severe thermal injury-induced remote acute lung injury in rats. *Burns* 41 1449–1458. 10.1016/J.Burns.2015.03.004 26188895

[B59] Straub R. H. (2004). Complexity of the bi-directional neuroimmune junction in the spleen. *Trends Pharmacol. Sci.* 25 640–646. 10.1016/J.Tips.2004.10.007 15530642

[B60] Tatewaki M. Harris M. Uemura K. Ueno T. Hoshino E. Shiotani A. (2003). Dual effects of acupuncture on gastric motility in conscious rats. *Am. J. Physiol. Regul. Integr. Comp. Physiol.* 285 R862–R872. 10.1152/Ajpregu.00715.2002 12959921

[B61] Tian D. Mo F. Cai X. Miao Z. Xiao F. Chang Y. (2018). Acupuncture relieves motion sickness via the IRβ-ERk1/2-dependent insulin receptor signalling pathway. *Acupunct. Med.* 36 153–161. 10.1136/Acupmed-2016-011202 29436382

[B62] Tjen-A-Looi S. C. Guo Z. L. Fu L. W. Longhurst J. C. (2016). Paraventricular nucleus modulates excitatory cardiovascular reflexes during electroacupuncture. *Sci. Rep.* 6:25910. 10.1038/Srep25910 27181844 PMC4867624

[B63] Tjen-A-Looi S. C. Guo Z. L. Li M. Longhurst J. C. (2013). Medullary gabaergic mechanisms contribute to electroacupuncture modulation of cardiovascular depressor responses during gastric distention in rats. *Am. J. Physiol. Regul. Integr. Comp. Physiol.* 304 R321–R332. 10.1152/Ajpregu.00451.2012 23302958 PMC3602723

[B64] Tjen-A-Looi S. C. Li P. Longhurst J. C. (2003). Prolonged inhibition of rostral ventral lateral medullary premotor sympathetic neurons by electroacupuncture in cats. *Auton. Neurosci.* 106 119–131. 10.1016/S1566-0702(03)00076-6 12878081

[B65] Tjen-A-Looi S. C. Li P. Longhurst J. C. (2006). Midbrain vlpag inhibits rvlm cardiovascular sympathoexcitatory responses during electroacupuncture. *Am. J. Physiol. Heart Circ. Physiol.* 290 H2543–H2553. 10.1152/Ajpheart.01329.2005 16428348

[B66] Tominaga K. Fujikawa Y. Tsumoto C. Kadouchi K. Tanaka F. Kamata N. (2016). Disorder of autonomic nervous system and its vulnerability to external stimulation in functional dyspepsia. *J. Clin. Biochem. Nutr.* 58 161–165. 10.3164/Jcbn.15-140 27013784 PMC4788403

[B67] Torres-Rosas R. Yehia G. Peña G. Mishra P. Del Rocio Thompson-Bonilla M. Moreno-Eutimio M. A. (2014). Dopamine mediates vagal modulation of the immune system by electroacupuncture. *Nat. Med.* 20 291–295. 10.1038/Nm.3479 24562381 PMC3949155

[B68] Ulloa L. (2005). The vagus nerve and the nicotinic anti-inflammatory pathway. *Nat. Rev. Drug Discov.* 4 673–684. 10.1038/Nrd1797 16056392

[B69] Ulloa L. (2021). Electroacupuncture activates neurons to switch off inflammation. *Nature* 598 573–574. 10.1038/D41586-021-02714-0 34646023 PMC9628694

[B70] Ulloa L. Quiroz-Gonzalez S. Torres-Rosas R. (2017). Nerve stimulation: Immunomodulation and control of inflammation. *Trends Mol. Med.* 23 1103–1120. 10.1016/J.Molmed.2017.10.006 29162418 PMC5724790

[B71] Ulloa L. (2022). Bioelectronic neuro-immunology: Neuronal networks for sympathetic-splenic and vagal-adrenal control. *Neuron* 10.1016/J.Neuron.2022.09.015 [Epub ahead of print]. 36202096

[B72] Van Houten J. M. Wessells R. J. Lujan H. L. Dicarlo S. E. (2015). My gut feeling says rest: Increased intestinal permeability contributes to chronic diseases in high-intensity exercisers. *Med. Hypotheses* 85 882–886. 10.1016/J.Mehy.2015.09.018 26415977

[B73] Wang H. Liu W. J. Shen G. M. Zhang M. T. Huang S. He Y. (2015). Neural mechanism of gastric motility regulation by electroacupuncture at RN12 And BL21: A paraventricular hypothalamic nucleus-dorsal vagal complex-vagus nerve-gastric channel PATHWAY. *World J. Gastroenterol.* 21 13480–13489. 10.3748/Wjg.V21.I48.13480 26730159 PMC4690177

[B74] Wang H. Shen G. M. Liu W. J. Huang S. Zhang M. T. (2013). The neural mechanism by which the dorsal vagal complex mediates the regulation of the gastric motility by weishu (RN12) and Zhongwan (BL21) stimulation. *Evid. Based Complement Alternat. Med.* 2013:291764. 10.1155/2013/291764 23843870 PMC3697139

[B75] Wang H. Yang G. Wang S. Zheng X. Zhang W. Li Y. (2018). The most commonly treated acupuncture indications in the United States: A cross-sectional study. *Am. J. Chin. Med* 7, 1–33. 10.1142/S0192415x18500738 30298749

[B76] Wang J. Wang Y. Q. Yu J. Cao X. D. Wu G. C. (2005). Electroacupuncture suppresses surgical trauma stress-induced lymphocyte apoptosis in rats. *Neurosci. Lett.* 383 68–72. 10.1016/J.Neulet.2005.03.068 15936514

[B77] Wang J. J. Ming Q. Liu X. D. Huang Y. X. Chen L. W. Qiu J. Y. (2007). Electro-acupuncture of foot yangming regulates gastric activity possibly through mediation of the dorsal vagal complex. *Am. J. Chin. Med.* 35 455–464. 10.1142/S0192415x07004977 17597504

[B78] Wehrwein E. A. Orer H. S. Barman S. M. (2016). Overview of the anatomy, physiology, and pharmacology of the autonomic nervous system. *Compr. Physiol.* 6 1239–1278. 10.1002/Cphy.C150037 27347892

[B79] Weng Z. J. Wu L. Y. Zhou C. L. Dou C. Z. Shi Y. Liu H. R. (2015). Effect of electroacupuncture on P2X3 receptor regulation in the peripheral and central nervous systems of rats with visceral pain caused by irritable bowel syndrome. *Purinergic Signal.* 11 321–329. 10.1007/S11302-015-9447-6 25809868 PMC4529849

[B80] Wu J. C. Ziea E. T. Lao L. Lam E. F. Chan C. S. Liang A. Y. (2010). Effect of electroacupuncture on visceral hyperalgesia, serotonin and fos expression in an animal model of irritable bowel syndrome. *J. Neurogastroenterol. Motil.* 16 306–314. 10.5056/Jnm.2010.16.3.306 20680170 PMC2912124

[B81] Yang N. N. Yang J. W. Ye Y. Huang J. Wang L. Wang Y. (2021). Electroacupuncture ameliorates intestinal inflammation by activating α7nachr-mediated JAK2/STAT3 signaling pathway in postoperative ileus. *Theranostics* 11 4078–4089. 10.7150/Thno.52574 33754049 PMC7977469

[B82] Yang S. Chang M. C. (2019). Chronic pain: Structural and functional changes in brain structures and associated negative affective states. *Int. J. Mol. Sci.* 20:3130. 10.3390/Ijms20133130 31248061 PMC6650904

[B83] Zhang C. R. Xia C. M. Jiang M. Y. Zhu M. X. Zhu J. M. Du D. S. (2013). Repeated electroacupuncture attenuating of apelin expression and function in the rostral ventrolateral medulla in stress-induced hypertensive rats. *Brain Res. Bull.* 97 53–62. 10.1016/J.Brainresbull.2013.05.013 23751198

[B84] Zhang M. Sun J. Wang Y. Tian Z. (2021). Secretagogin mediates the regulatory effect of electroacupuncture on hypothalamic-pituitary-adrenal axis dysfunction in surgical trauma. *Neural Plast.* 2021:8881136. 10.1155/2021/8881136 33628224 PMC7880713

[B85] Zhang Q. Tan Y. Wen X. Li F. (2022). Involvement of neuropeptide y within paraventricular nucleus in electroacupuncture inhibiting sympathetic activities in hypertensive rats. *Int. J. Hypertens* 2022:9990854. 10.1155/2022/9990854 35087687 PMC8789434

[B86] Zhang Q. Tan Y. Y. Liu X. H. Yao F. R. Cao D. Y. (2018). Electroacupuncture improves baroreflex and γ-aminobutyric acid type b receptor-mediated responses in the nucleus tractus solitarii of hypertensive rats. *Neural Plast.* 2018:8919347. 10.1155/2018/8919347 30363902 PMC6186317

[B87] Zhang R. Lao L. Ren K. Berman B. M. (2014). Mechanisms of acupuncture-electroacupuncture on persistent pain. *Anesthesiology* 120 482–503. 10.1097/Aln.0000000000000101 24322588 PMC3947586

[B88] Zhang S. Li S. Liu Y. Ye F. Yin J. Foreman R. D. (2018). Electroacupuncture via chronically implanted electrodes improves gastric dysmotility mediated by autonomic-cholinergic mechanisms in a rodent model of functional dyspepsia. *Neurogastroenterol. Motil.* 30:E13381. 10.1111/Nmo.13381 29856090

[B89] Zhang X. F. Xiang S. Y. Geng W. Y. Cong W. J. Lu J. Jiang C. W. (2018). Electro-acupuncture regulates the cholinergic anti-inflammatory pathway in a rat model of chronic obstructive pulmonary disease. *J. Integr. Med.* 16 418–426. 10.1016/J.Joim.2018.10.003 30341024

[B90] Zhang X. H. Feng C. C. Pei L. J. Zhang Y. N. Chen L. Wei X. Q. (2021). Electroacupuncture attenuates neuropathic pain and comorbid negative behavior: The involvement of the dopamine system in the amygdala. *Front. Neurosci.* 15:657507. 10.3389/Fnins.2021.657507 34025342 PMC8137986

[B91] Zhao L. Chen J. Li Y. Sun X. Chang X. Zheng H. (2017). The long-term effect of acupuncture for migraine prophylaxis: A randomized clinical trial. *JAMA Intern. Med.* 177 508–515. 10.1001/Jamainternmed.2016.9378 28241154

[B92] Zhao M. Wang Z. Weng Z. Zhang F. Li G. Ma Z. (2020). Electroacupuncture improves ibs visceral hypersensitivity by inhibiting the activation of astrocytes in the medial thalamus and anterior cingulate cortex. *Evid. Based Complement Alternat. Med.* 2020:2562979. 10.1155/2020/2562979 32617101 PMC7306073

[B93] Zhou H. Liang H. Li Z. F. Xiang H. Liu W. Li J. G. (2013). Vagus nerve stimulation attenuates intestinal epithelial tight junctions disruption in endotoxemic mice through A 7 nicotinic acetylcholine receptors. *Shock* 40 144–151. 10.1097/Shk.0b013e318299e9c0 23860583

[B94] Zhou W. Fu L. W. Guo Z. L. Longhurst J. C. (2007). Role of glutamate in the rostral ventrolateral medulla in acupuncture-related modulation of visceral reflex sympathoexcitation. *Am. J. Physiol. Heart Circ. Physiol.* 292 H1868–H1875. 10.1152/Ajpheart.00875.2006 17158649

